# Continuous CO_2_ capture and methanation over Ni–Ca/Al_2_O_3_ dual functional materials†

**DOI:** 10.1039/d2ra07554g

**Published:** 2023-01-12

**Authors:** Lingcong Li, Ziyang Wu, Shinta Miyazaki, Takashi Toyao, Zen Maeno, Ken-ichi Shimizu

**Affiliations:** a Institute for Catalysis, Hokkaido University N-21, W-10 Sapporo 001-0021 Japan kshimizu@cat.hokudai.ac.jp; b School of Advanced Engineering, Kogakuin University 2665-1, Nakano-cho Hachioji 192-0015 Japan zmaeno@cc.kogakuin.ac.jp

## Abstract

Although Ni–Ca-based dual functional materials (DFMs) have been examined for CO_2_ capture and reduction with H_2_ (CCR) for the synthesis of CH_4_, their performance has generally been investigated using single reactors in an oxygen-free environment. In addition, continuous CCR operations have scarcely been investigated. In this study, continuous CCR for the production of CH_4_ was investigated using a double reactor system over Al_2_O_3_-supported Ni–Ca DFMs in the presence of O_2_. We found that a high Ca loading (Ni(10)–Ca(30)/Al_2_O_3_, 10 wt% Ni, and 30 wt% CaO) was necessary for reaction efficiency under isothermal conditions at 450 °C. The optimized DFM exhibited an excellent performance (46% CO_2_ conversion, 45% CH_4_ yield, and 97% CH_4_ selectivity, respectively) and good stability over 24 h. The structure and CCR activity of Ni(10)–Ca(30)/Al_2_O_3_ were studied using X-ray diffraction (XRD), scanning transmission electron microscopy (STEM), energy-dispersive X-ray spectrometry (EDS), temperature-programmed desorption (TPD), and temperature-programmed surface reaction (TPSR) techniques.

## Abstract 2

Continuous CO_2_ capture and reduction with H_2_ for the synthesis of CH_4_ was investigated using a double reactor system over Al_2_O_3_-supported Ni–Ca dual functional materials in the presence of O_2_.

## Graphical abstract

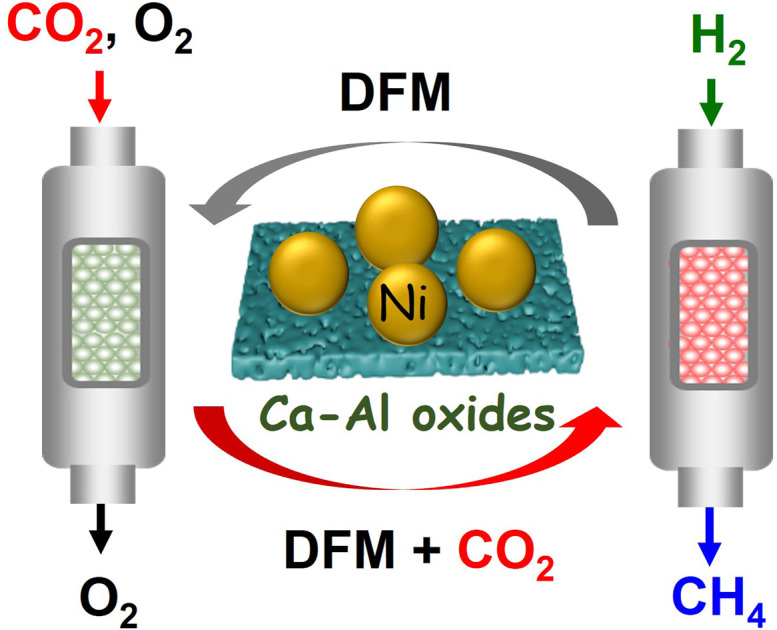

## Introduction

As a major component of greenhouse gases, CO_2_ is a significant contributor to global warming. To reduce CO_2_ emissions and establish a low-carbon society, carbon capture and utilization (CCU) strategies, including CO_2_ capture and reduction (CCR) using H_2_ or hydrocarbons, provide valuable approaches.^1^ Among these, CO_2_ capture and methanation is an effective protocol for the synthesis of carbon-neutral CH_4_.^2^ Alkali and alkaline earth metals are widely used for CO_2_ capture, owing to their high capacity for CO_2_ uptake and the ability to form metal carbonates. To promote carbonate hydrogenation, transition metals, such as Ru, Pt, Fe, Ni, and Cu are utilized. Thus, dual functional materials (DFMs) consisting of alkali/earth alkaline metal salts and transition metal species have attracted considerable interest in recent years.^3^

CaO is generally used for CO_2_ capture, and Ni is particularly promising owing to its low price.^4^ Therefore, Ni–Ca DFMs hold promise for CO_2_ capture and methanation processes. For example, Yang *et al.*^5^ found that CO_2_ conversion and CH_4_ selectivity were higher with the use of Ni/CaO–Al_2_O_3_ than with that of Ni/Al_2_O_3_ as a result of surface coverage by CO_2_-derived species on the CaO–Al_2_O_3_ surface. Alipour *et al.*^6^ reported that the capacity for CO_2_ capture was improved noticeably when CaO, MgO, or BaO was loaded onto a Ni/Al_2_O_3_ catalyst. Bermejo-Lopez *et al.*^4^ investigated the effect of Ni content in DFMs on CCR using CaO as a sorbent. They established that the maximum CH_4_ formation (142 μmol g^−1^) at 520 °C was achieved using 15Ni15Ca (15 wt% Ni, and 15 wt% CaO). Sun *et al.*^7^ studied the effect of the interactions between the Ni active sites and CaO sorbents on the CCR process. They found that CO_2_ conversion and CH_4_ selectivity using 1% Ni/CeO_2_–CaO, obtained by physical mixing of 1% Ni/CeO_2_ and CaO in a mass ratio of 1 : 1, increased significantly to 62% and 84%, respectively, when the distance between the catalytic sites and sorbents was increased to a suitable scale. Although CCR over DFMs aims to utilize diluted CO_2_ in air or flue gas, most previous studies on Ni-based DFMs were conducted under O_2_-free conditions.^4–7^ Recently, Kuramoto and co-workers^8^ studied the CCR activity of Ni-based DFMs comprising Na, K, or Ca, in the presence of oxygen. They found that when the operational pressure was increased from 0.1 to 0.9 MPa using 400 ppm CO_2_, CH_4_ production over Ni–Na/Al_2_O_3_ increased from 111 to 160 μmol g^−1^. Although pressure elevation effectively enhances the CCR performance, mild reaction conditions are preferable from an economic viewpoint.

Recently, we developed DFMs comprising Na-modified Pt NPs on Al_2_O_3_ as effective DFMs for CO_2_ capture in the presence of O_2_ and reduction with H_2_ to generate CO.^9^ A continuous CCR operation was achieved using a double reactor system, whereby the valves on the top and bottom were controlled to switch the gas supply. This protocol was proposed by Urakawa *et al.* in the first time.^10^ In this study, continuous CCR was investigated using Al_2_O_3_-supported Ni–Ca DFMs. We optimized the loading amounts of Ni and Ca and found that a high Ca loading (Ni = 10 wt% and CaO = 30 wt%, Ni(10)–Ca(30)/Al_2_O_3_) was optimal for CCR under isothermal conditions at 450 °C. Characterization of the high Ca-content Ni–Ca/Al_2_O_3_ DFM revealed the formation of a Ca–Al mixed oxide phase derived from the mayenite (Ca_12_Al_14_O_33_) structure.^11^ The impact of mixed oxide formation on CO_2_ adsorption and desorption, as well as on the hydrogenation of adsorbed CO_2_ was discussed.

## Experimental

### DFM preparation

Ni–Ca/Al_2_O_3_ DFMs were synthesized using the wetness impregnation method. The γ-Al_2_O_3_ support was obtained by calcination of boehmite (γ-AlOOH, Sasol Chemicals) at 900 °C for 3 h. An appropriate amount of aqueous Ca(NO_3_)_2_·H_2_O (AR 98.5%, FUJIFILM Wako Pure Chemical Corporation) was stirred for 3 h with calcined γ-Al_2_O_3_ for impregnation (Ca: 6, 20, 30, 40, and 50 wt%). The resultant suspension of Al_2_O_3_ and Ca(NO_3_)_2_ was then evaporated at 50 °C using a vacuum pump, followed by drying at 100 °C overnight. The Ca/Al_2_O_3_ support was obtained after calcination at 600 °C for 2 h. Next, Ca–Al_2_O_3_ was impregnated with Ni(NO_3_)_2_·6H_2_O (AR > 99%, FUJIFILM Wako Pure Chemical Corporation) (Ni: 1, 5, 10, and 15 wt%) as follows. A Ca/Al_2_O_3_ suspension in Ni precursor solution was stirred at room temperature for 30 min, and then the mixture was evaporated, dried, and calcined, as described for the Ca–Al_2_O_3_ support preparation procedure. Finally, Ni–Ca/Al_2_O_3_ DFMs with varying Ca loadings were obtained, denoted as Ni(*x*)–Ca(*y*)/Al_2_O_3_ (where *x* and *y* are the loadings of Ni and Ca, respectively).

For the synthesis of Ca_12_Al_14_O_33_, mixed aqueous solutions of Ca(NO_3_)_2_·H_2_O and γ-AlOOH in a molar ratio of 12 : 7 were continuously stirred at room temperature for 3 h and then evaporated at 50 °C using a vacuum pump, followed by drying at 100 °C overnight. The dried powder was calcined at 1050 °C for 2 h to obtain Ca_12_Al_14_O_33_. Finally, Ni/Ca_12_Al_14_O_33_ was obtained using the above-described procedure for the synthesis of Ni–Ca/Al_2_O_3_ DFMs.

### Characterization

Powder X-ray diffraction (XRD) measurements were carried out on a Rigaku MiniFlex II/AP diffractometer with Cu Kα radiation. High-Angle Annular Dark Field Scanning Transmission Electron Microscopy (HAADF-STEM) images were recorded on a FEI Titan G2 microscope equipped with an energy dispersive X-ray (EDX) analyzer. The specific surface area was calculated using Brunauer–Emmet–Teller (BET) theory over the range *P*/*P*_0_ = 0.1–0.3. Temperature programmed desorption of carbon dioxide (CO_2_ TPD) was performed on a vertical quartz fixed-bed flow reactor connected with a mass spectrometer (Microtrac BEL Corp.). A 100 mg of sample was put on quartz wool in the middle of the reactor. The reactor set in an electric tube furnace and purged under N_2_ flow (95 mL min^−1^) for 30 min at 450 °C, and then cool down the sample to room temperature. Next, the 1% CO_2_/N_2_ (100 mL min^−1^) mixed gases fed into the reactor for 30 min and then flowed N_2_ for 15 min. After that, the TPD profile was obtained by heating the sample from 30 to 650 °C in N_2_ flow with elevating temperature by 10 °C min^−1^. Temperature program surface reaction (TPSR) was performed on the same equipment as described for TPD measurement. First, sample with 100 mg was put on quartz wool in the middle of the reactor. The reactor set in an electric tube furnace and pretreatment under 10% H_2_/N_2_ flow (100 mL min^−1^) for 30 min at 450 °C. The sample was cooled down to room temperature in subsequently. Next, 1% CO_2_/10% O_2_/N_2_ (100 mL min^−1^) flow was fed into the reactor for 30 min and then flowed N_2_ for 15 min. Finally, TPSR profile was obtained by heating the sample from 30 to 650 °C by 10 °C min^−1^ with 5% H_2_/N_2_ flow (100 mL min^−1^).

### Continuous CCR operation

**Fig. 1:**
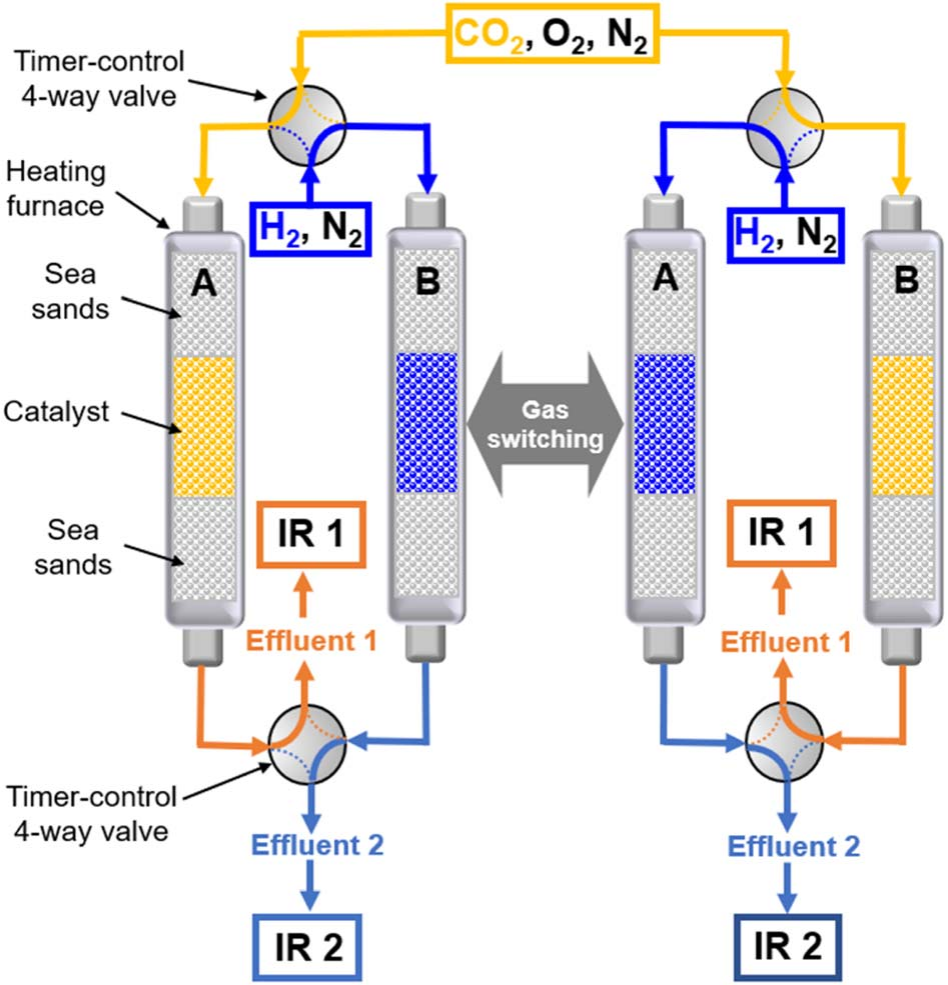
Diagram of the continuous-operation CCR double reactor system.

CCR was performed in continuous separated fixed-bed flow reactors (Fig. 1). The similar reaction system has been developed in our previous study.^9^ Two vertical quartz reactors were utilized (reactors A and B), and 100 mg of the sample was placed on quartz wool in the middle of the reactor. Two sides of the tube were filled with sea sand. Both reactors were equipped with the same DFM and placed in an electric tube furnace. Reactors were heated to 500 °C under N_2_ flow (90 mL min^−1^), followed by the introduction of 10% H_2_/N_2_ (100 mL min^−1^) for pretreatment over 20 min at 500 °C. The two timer-control 4-way valves were switched simultaneously to continuously collect the effluent gases containing uncaptured CO_2_ (effluent 1) and generated CO and CH_4_, and desorbed CO_2_ (effluent 2) from each outlet line. The compositions of effluents 1 and 2 were monitored employing Fourier transform infrared (FTIR) spectroscopy (JASCO FT/IR-4600) using gas cells. The background spectrum was acquired once the temperature decreased to 450 °C after H_2_ pretreatment followed by N_2_ purging. Next, 100 mL min^−1^ of 1% CO_2_/10% O_2_/N_2_ was fed to reactor A for 30 s, and then the gas feed was switched to 100 mL min^−1^ of pure H_2_ for 30 s. Reactor B underwent reverse treatment; thus, 100 mL min^−1^ of pure H_2_ was fed into reactor B for 30 s, and then the gas was switched to 100 mL min^−1^ of 1% CO_2_/10% O_2_/N_2_ for 30 s. CCR was also conducted using a single reactor for comparison. Thus, only one reactor was equipped with a DFM (100 mg) and the other reactor was filled with pure sea sand. The subsequent steps implemented for the single reactor were the same as those described for the continuous operation of the double reactor system. The CO_2_ capture amount (*q*_CO_2__), amount of generated CO and CH_4_ (denoted as *q*_CO_, *q*_CH_4__, respectively), selectivity for CO (Sel_CO_), and conversion of captured CO_2_ (Conv_capCO_2__) were calculated as follows:1
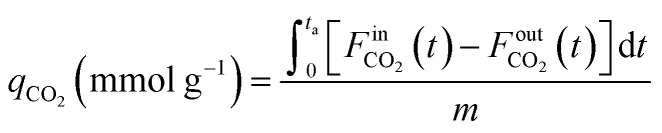
2
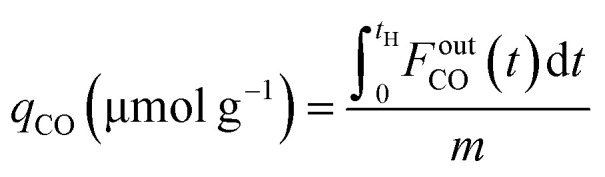
3
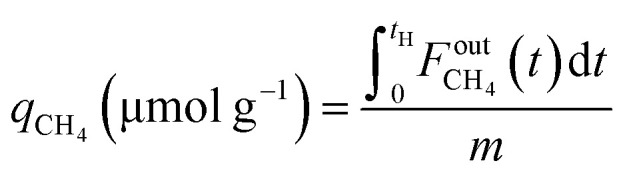
4
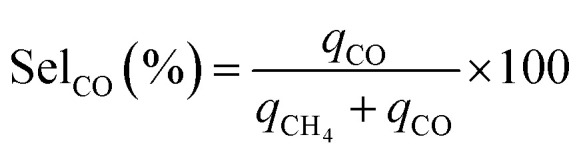
5
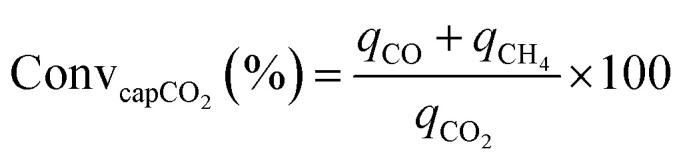
where 
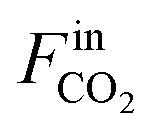
 and 
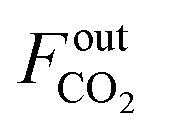
 are the CO_2_ molar flow rates at the column inlet and outlet, respectively; *F*^out^_CO_ and 
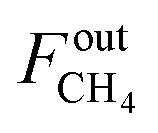
 are the CO and CH_4_ molar flow rates at the column outlet, respectively; *m* is the DFM mass; *t*_a_ is the duration of the adsorption stage; *t*_H_ denotes the duration of the H_2_ reduction process.

## Results and discussion

**Fig. 2:**
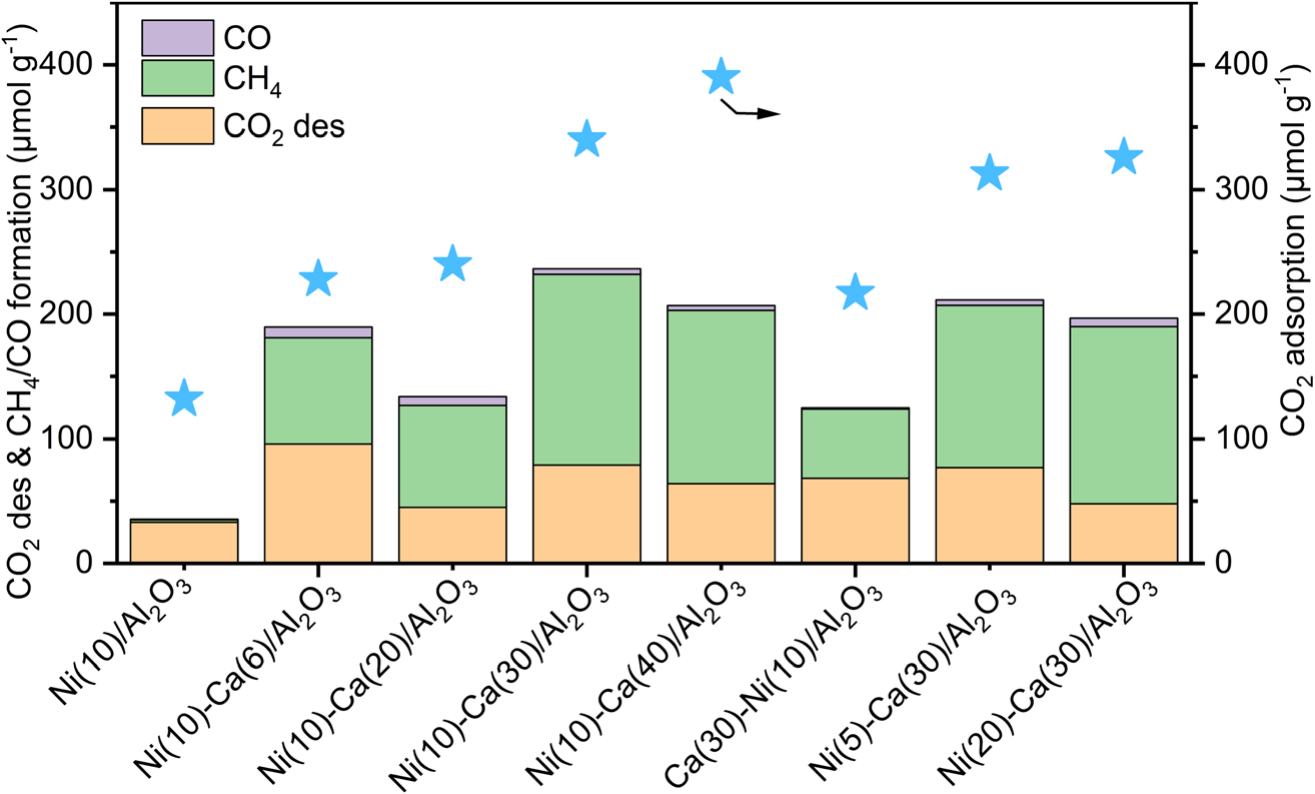
Effluent gas compositions for continuous CCR operation over Ni(*x*)–Ca(*y*)/Al_2_O_3_ with varying Ca and Ni loadings. Conditions: 100 mg sample for each reactor, 450 °C, 100 mL min^−1^ of 1% CO_2_/10% O_2_/N_2_ for 30 s, then switched to 100 mL min^−1^ of H_2_ for 30 s.

DFMs comprising different loading amounts of Ni and Ca on Al_2_O_3_ were screened to determine the optimal composition for the continuous CCR system. First, the effect of Ca content was studied, while the Ni loading was kept constant at 10 wt% (CaO: 0, 6, 20, 30, and 40 wt%). As shown in Fig. 2, Ni(10)/Al_2_O_3_ without Ca adsorbed a moderate amount of CO_2_ (132 μmol g^−1^), but CH_4_ production was low (2 μmol g^−1^). The CO_2_ capture amount gradually increased from 228 to 390 μmol g^−1^ as the Ca content increased from 6 to 40 wt% whereas CH_4_ formation was the highest (153 μmol g^−1^) for Ni(10)–Ca(30)/Al_2_O_3_. This result illustrates the importance of optimizing the Ca loading on Ni–Ca/Al_2_O_3_ DFMs. The CO_2_ conversion, CH_4_ yield, and CH_4_ selectivity in CCR using Ni(10)–Ca(30)/Al_2_O_3_ were 46%, 45%, and 97%, respectively. These values were comparable with those for reported Ni–Ca DFMs (Table S1†). Next, Ni(*x*)–Ca(30)/Al_2_O_3_ containing varying Ni loadings (Ni: 5, 10, and 20 wt%) were also prepared and tested for continuous CCR (Fig. 2). A Ni loading of 10 wt% was found to be optimal for both CO_2_ adsorption and CH_4_ formation. Thus, the optimal loading amounts of Ca and Ni were 30 and 10 wt%, respectively, for continuous CCR operation under isothermal conditions at 450 °C. Furthermore, 30 wt% CaO was loaded onto Ni(10)/Al_2_O_3_ and then applied to continuous CCR to investigate the effect of the Ni and Ca loading sequence (Fig. 2). The amounts of CO_2_ captured and CH_4_ formed over Ca(30)–Ni(10)/Al_2_O_3_ were 217 and 64 μmol g^−1^, respectively. These values are lower than those for Ni(10)–Ca(30)/Al_2_O_3_ (340 and 153 μmol g^−1^), suggesting the importance of the loading sequence of Ni and Ca onto Al_2_O_3_. Thus, Ni(10)–Ca(30)/Al_2_O_3_ was applied in further investigations in this study.

Next, the comparison of continuous CCRs using double and single reactor systems using Ni(10)–Ca(30)/Al_2_O_3_ is shown in Fig. S1.† The effluent concentration profiles of uncaptured CO_2_, generated CH_4_ and CO, and desorbed CO_2_ during continuous CCR are shown in Fig. S1a.† The concentration of uncaptured CO_2_ ranged from 1100 to 2200 ppm. The highest concentration of formed CH_4_ was 4600 ppm, with a selectivity of 97%, and the amount of CO formed was <150 ppm. The concentration of desorbed CO_2_ ranged from 1300 to 2300 ppm. In the single reactor system containing Ni(10)–Ca(30)/Al_2_O_3_ (Fig. S1b†), the concentration of uncaptured CO_2_ (100–7500 ppm) was higher than that in the double reactors. The desorbed CO_2_ in the single reactor ranged from 200 to 2700 ppm, whereas the highest concentrations of CH_4_ (680 ppm) and CO (40 ppm) were considerably lower than those in the double reactors. These results indicate that CO_2_ capture and methanation efficiency were significantly improved using a double reactor system.

**Fig. 3:**
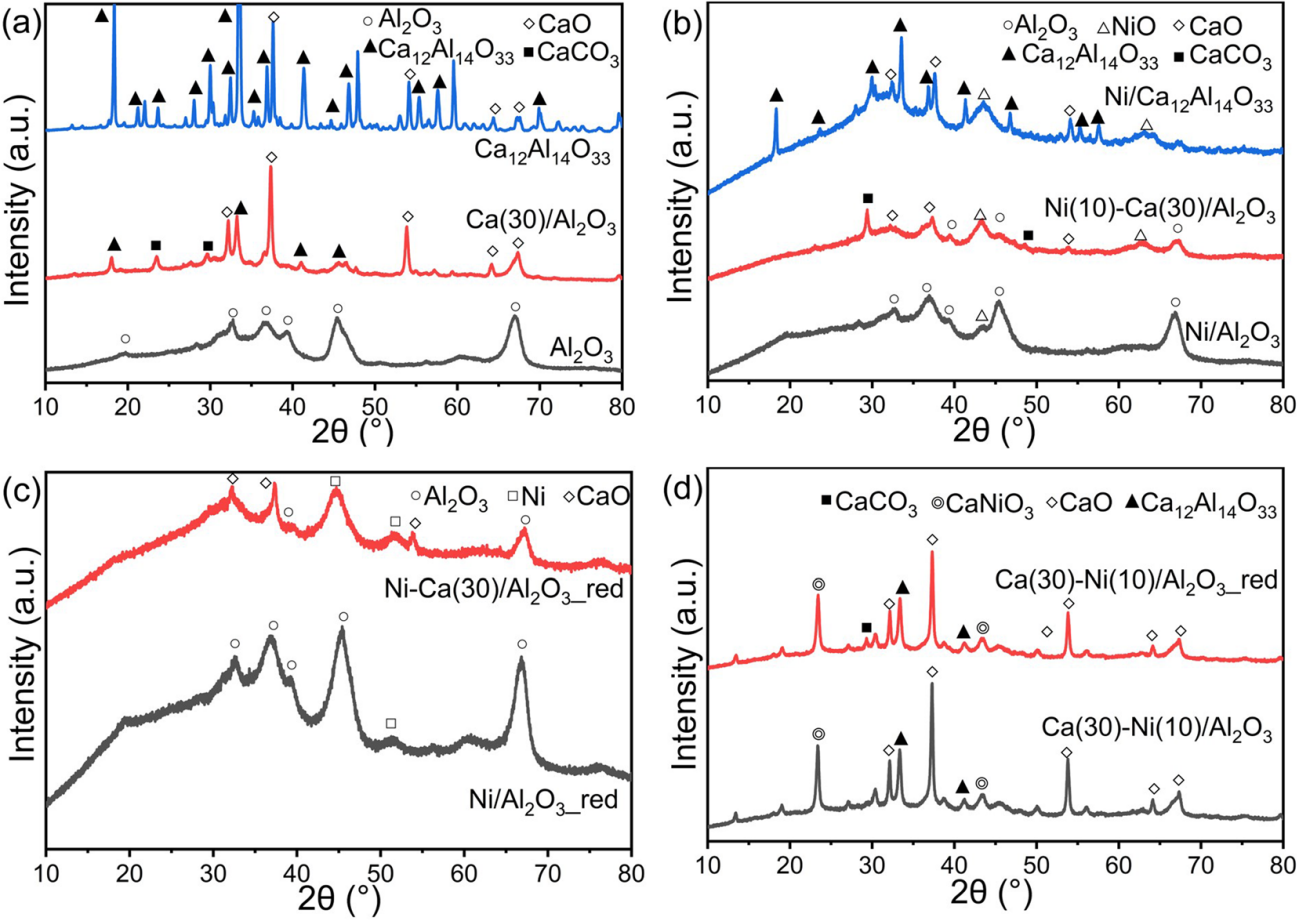
XRD patterns of (a) Al_2_O_3_, Ca(30)/Al_2_O_3_, and Ca_12_Al_14_O_33_ without Ni loading; (b) calcined Ni(10)/Al_2_O_3_, Ni(10)–Ca(30)/Al_2_O_3_ and Ni/Ca_12_Al_14_O_33_; (c) reduced Ni(10)/Al_2_O_3_ and Ni(10)–Ca(30)/Al_2_O_3_; (d) calcined and reduced Ca(30)–Ni(10)/Al_2_O_3_.

Fig. 3a shows XRD patterns of Al_2_O_3_ and Ca(30)/Al_2_O_3_ without Ni. Peaks at 19.6°, 31.9°, 37.6°, 39.5°, 45.8°, 60.5°, and 66.8° were observed for the Al_2_O_3_ support, assignable to the γ-Al_2_O_3_ phase (JCPDS No. 50-0741). For Ca(30)/Al_2_O_3_, peaks arising from the CaO (JCPDS No. 37-1497) and CaCO_3_ (JCPDS No. 17-0763) phases were observed. In addition, the diffraction pattern of Ca_12_Al_14_O_33_ (mayenite, JCPDS No. 48-1882) appeared, and the γ-Al_2_O_3_ phase almost disappeared, suggesting that γ-Al_2_O_3_ was possibly converted to Ca_12_Al_14_O_33_. A pure Ca_12_Al_14_O_33_ phase was also synthesized using calcium nitrate and boehmite as precursors in a suitable stoichiometric ratio and calcination at 1050 °C. The XRD patterns indicated that the prepared sample largely comprised Ca_12_Al_14_O_33_ (Fig. 3a) and a very small amount of CaO. This verified the formation of the Ca_12_Al_14_O_33_ structure over Ca(30)/Al_2_O_3_. Fig. 3b shows the XRD patterns of Ni-loaded Al_2_O_3_, Ca(30)/Al_2_O_3_, and Ca_12_Al_14_O_33_. For calcined Ni(10)/Al_2_O_3_, in addition to the Al_2_O_3_ peaks, a peak appeared at 43.3°, which was attributed to the (200) facet of NiO (JCPDS No. 47-1049). A comparison of the XRD patterns of Ni(10)/Al_2_O_3_ and Ni(10)–Ca(30)/Al_2_O_3_ revealed that the intensity of the NiO peak was higher for the latter, indicating that Ca-loaded Al_2_O_3_ promoted NiO crystal growth. Notably, the Ca_12_Al_14_O_33_ phase was not detected after Ni loading, whereas the CaO and CaCO_3_ phases were still present in Ni(10)–Ca(30)/Al_2_O_3_. For Ni(10)/Ca_12_Al_14_O_33_, the diffraction peaks assignable to the Ca_12_Al_14_O_33_ phase decreased dramatically, and a new phase did not appear. These results suggest that Ni loading likely induced the transformation of the Ca_12_Al_14_O_33_ phase to amorphous structures. After H_2_ reduction at 500 °C, the Ni metal phase (JCPDS No. 04-0850) appeared in both Ni(10)/Al_2_O_3_ and Ni(10)–Ca(30)/Al_2_O_3_ (Fig. 3c), indicating that NiO was converted to Ni metal under H_2_ flow. When 10 wt% of Ni was supported on Al_2_O_3_ first, followed by the introduction of 30 wt% of CaO onto Ni/Al_2_O_3_ Ca(30–Ni(10)/Al_2_O_3_), a diffraction pattern indicative of CaNiO_3_ (ID: mvc-3998) was observed with peaks assignable to CaO, CaCO_3_, and Ca_12_Al_14_O_33_ phases (Fig. 3d). The CaNiO_3_ phase was maintained after H_2_ treatment at 500 °C, and peaks attributable to Ni metal did not appear, suggesting that Ca–Ni composite oxides are difficult to reduce to Ni metal at CCR operation temperature (450 °C). The formation of CaNiO_3_ possibly led to a decrease in the amount of Ni active species, resulting in the inferior CCR performance of Ca(30)–Ni(10)/Al_2_O_3_ compared to that of Ni(10)–Ca(30)/Al_2_O_3_.

**Fig. 4:**
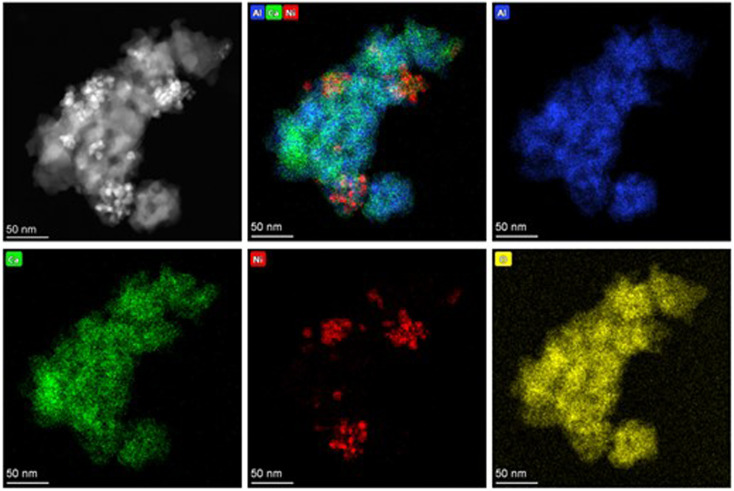
STEM images and EDS mapping of (a) Ni(10)/Al_2_O_3_ and (b) Ni(10)–Ca(30)/Al_2_O_3_.

The specific surface areas of Ni(10)/Al_2_O_3_, Ni(10)–Ca(30)/Al_2_O_3_, and Ca(30)/Al_2_O_3_ were 154, 37, and 34 m^2^ g^−1^, respectively. Ni(10)–Ca(30)/Al_2_O_3_ and Ca(30)/Al_2_O_3_ have similar specific surface areas, which are appreciably lower than that of Ni(10)/Al_2_O_3_. This is possibly due to the structural transformation of γ-Al_2_O_3_ to Ca_12_Al_14_O_33_ phase. The similar surface area values were (20–40 m^2^ g^−1^) obtained in the solid-phase synthesis of Ca_12_Al_14_O_33_ in the previous literature.^12^ The morphology and distribution of the Ni nanoparticles on Ni(10)/Al_2_O_3_ and Ni(10)–Ca(30)/Al_2_O_3_ were characterized using STEM and EDS mapping (Fig. S2† and 4). In the case of Ni(10)/Al_2_O_3_, the Ni nanoparticles were dispersed on Al_2_O_3_ with an average size of 6.9 nm (Fig. S2†), whereas for Ni(10)–Ca(30)/Al_2_O_3_, the Ni nanoparticles were aggregated and the particle size increased (Fig. 4), which is consistent with the XRD results.

**Fig. 5:**
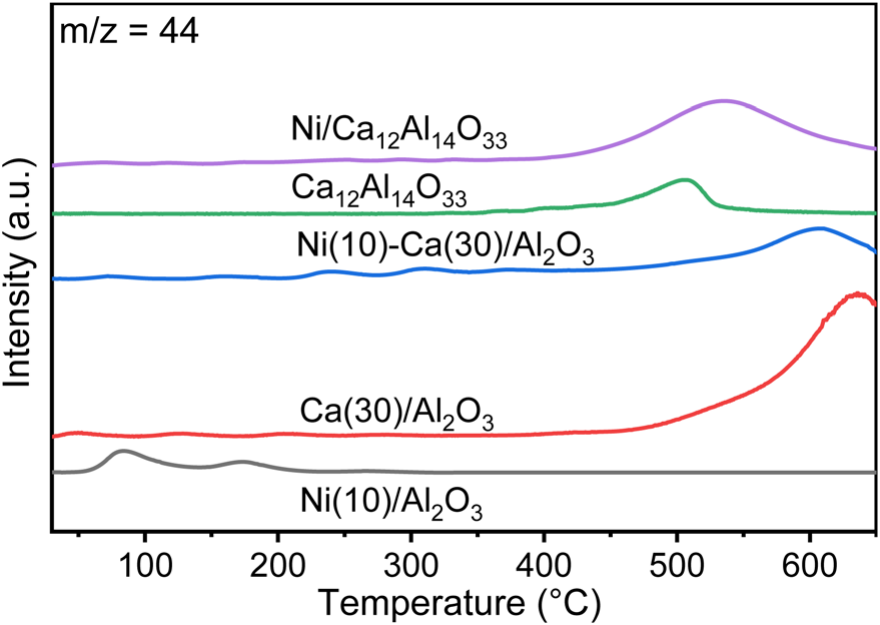
CO_2_ TPD profiles for Ni(10)/Al_2_O_3_, Ca(30)/Al_2_O_3_, Ni(10)–Ca(30)/Al_2_O_3_, Ca_12_Al_14_O_33_, and Ni(10)/Ca_12_Al_14_O_33_. Conditions: 100 mg of DFM, N_2_ pretreatment at 450 °C for 30 min, followed by cooling down to room temperature and capture using 1% CO_2_/N_2_ (100 mL min^−1^), and then an increase in temperature to 650 °C under pure N_2_ (100 mL min^−1^).

CO_2_ TPD measurements were conducted to explore the CO_2_ desorption properties of Ni(10)/Al_2_O_3_, Ca(30)/Al_2_O_3_, Ni(10)–Ca(30)/Al_2_O_3_, Ca_12_Al_14_O_33_, and Ni(10)/Ca_12_Al_14_O_33_ (Fig. 5). Ni(10)/Al_2_O_3_ gave rise to two desorption peaks at 80 and 160 °C, possibly arising from the physical adsorption of CO_2_ onto the surface of Al_2_O_3_. In contrast, the TPD profiles for Ni(10)–Ca(30)/Al_2_O_3_ and Ca(30)/Al_2_O_3_ displayed a dominant CO_2_ desorption peak during the temperature increase from 400 to 600 °C. A similar desorption peak was observed for Ni(10)/Ca_12_Al_14_O_33_ and Ca_12_Al_14_O_33_, implying that this CO_2_ desorption was derived from adsorbed CO_2_ over Ca–Al mixed oxides.

**Fig. 6:**
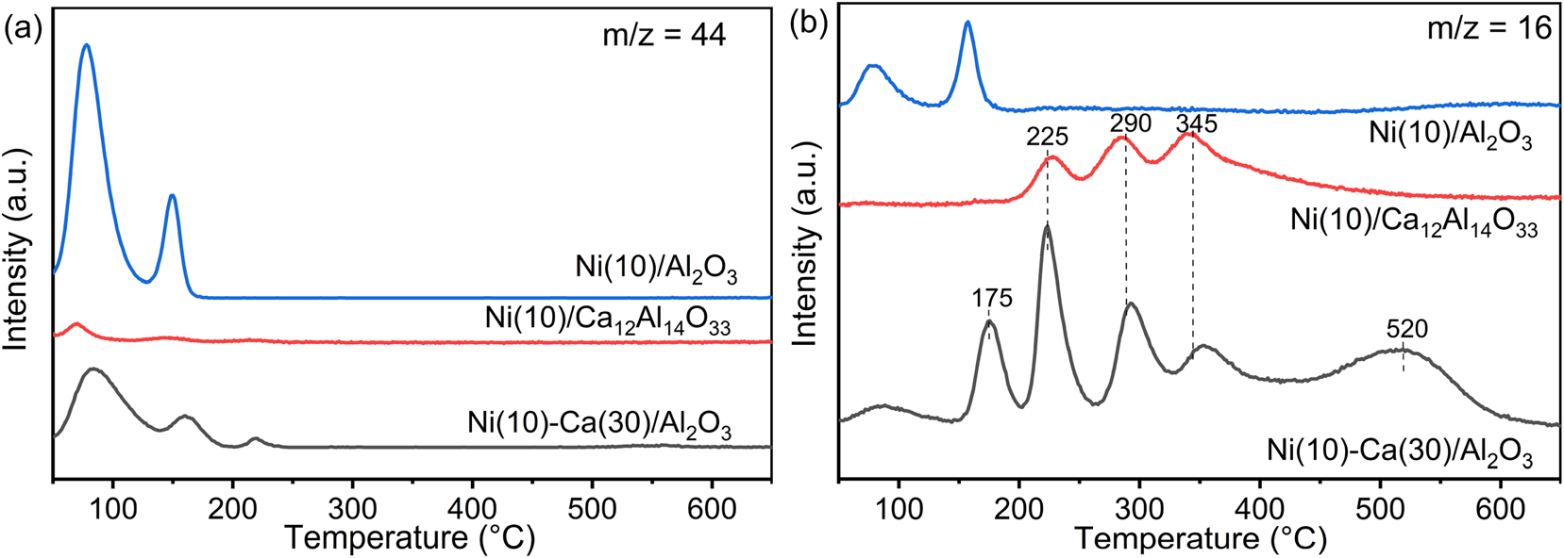
TPSR profiles of (a) CO_2_ and (b) CH_4_ for Ni(10)/Al_2_O_3_, Ni(10)–Ca(30)/Al_2_O_3_, and Ni (10)/Ca_12_Al_14_O_33_. Conditions: 100 mg of DFM, H_2_ pretreatment at 450 °C for 30 min, followed by cooling to room temperature and capture under 1% CO_2_/10% O_2_/N_2_ (100 mL min^−1^), and then an increase in temperature to 650 °C under 5% H_2_/N_2_ (100 mL min^−1^).

TPSR measurements were conducted using Ni(10)/Al_2_O_3_, Ni(10)–Ca(30)/Al_2_O_3_, and Ni(10)/Ca_12_Al_14_O_33_ to obtain insights into the formation of CH_4_ through the reduction of adsorbed CO_2_ with H_2_ (Fig. 6). Fig. 6a shows the CO_2_ desorption peaks during reduction with H_2_. Compared to the CO_2_ TPD profiles (Fig. 5), the temperature for the CO_2_ desorption peaks over Ni(10)/Al_2_O_3_ was similar to that of the CO_2_ adsorption peaks, while for Ni(10)–Ca(30)/Al_2_O_3_ and Ni(10)/Ca_12_Al_14_O_33_, the CO_2_ desorption peaks were nearly absent at temperatures above 400 °C. Fig. 6b shows the CH_4_ (*m*/*z* = 16) formation profiles. For Ni(10)/Al_2_O_3_, the CH_4_ formation temperature was similar to that of CO_2_ desorption, implying that once the adsorbed CO_2_ species were desorbed, the desorbed CO_2_ was converted to CH_4_ through reduction with H_2_. For Ni(10)–Ca(30)/Al_2_O_3_ and Ni(10)/Ca_12_Al_14_O_33_, the CH_4_ formation peaks were clearly observed at 225, 290, and 345 °C, being lower than the temperature range for CO_2_ desorption in their CO_2_ TPD profiles. This strongly indicates that the adsorbed CO_2_ species were directly converted to CH_4_. Additional CH_4_ formation peaks were observed at 175 and 520 °C for Ni(10)–Ca(30)/Al_2_O_3_. The CH_4_ formation peak below 200 °C resulted from the activity of Ni supported on Al_2_O_3_, and the peak at 520 °C possibly appeared due to the Ni supported on calcium oxide or carbonate species. Considering the CCR operation temperature of 450 °C, the Ni species supported on amorphous Ca–Al mixed oxides most likely played the dominant role in the reaction. The performance of Ni/Ca_12_Al_14_O_33_ in continuous CCR verified this hypothesis, whereby high CO_2_ capture and CH_4_ formation concentrations were obtained (Fig. S3†).

**Fig. 7:**
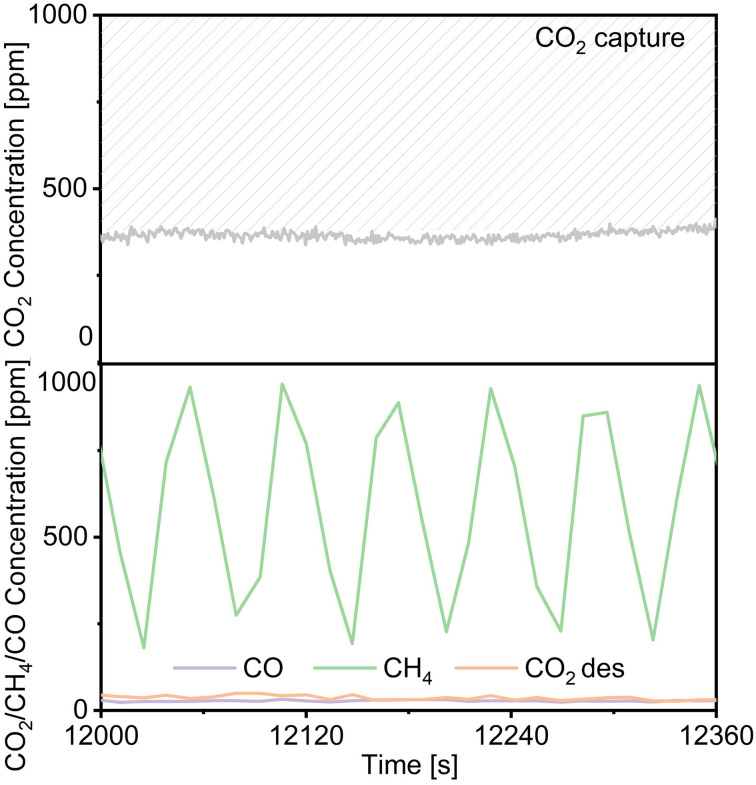
Effluent gas composition during continuous CCR operation over Ni(10)–Ca(30)/Al_2_O_3_ using a decreased CO_2_ concentration of 1000 ppm in the gas mixture. Conditions: 100 mg of DFM, 450 °C, 100 mL min^−1^ of 0.1% CO_2_/10% O_2_/N_2_ for 30 s, switched to 100 mL min^−1^ of H_2_ for 30 s.

**Fig. 8:**
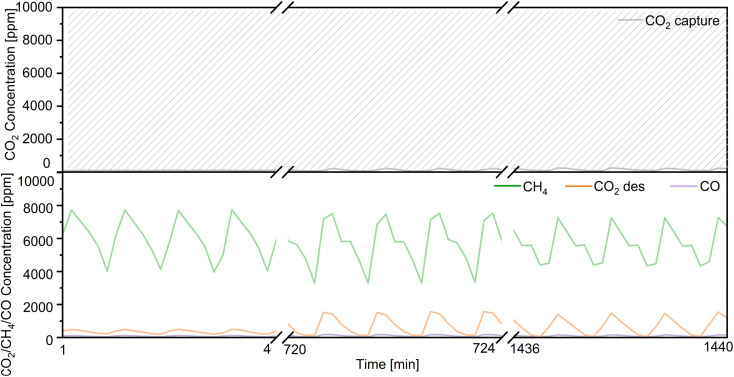
Long-term continuous CCR operation using the double reactor system for 24 h. Conditions: 300 mg of Ni(10)–Ca(30)/Al_2_O_3_ for each reactor, 450 °C, 100 mL min^−1^ of 1% CO_2_/10% O_2_/N_2_ for 30 s, switched to 100 mL min^−1^ of H_2_ for 30 s.

Regarding the utilization of low-concentration CO_2_, we decreased the flow rate of CO_2_ in the mixed gases from 1% CO_2_/10% O_2_/N_2_ to 0.1% CO_2_/10% O_2_/N_2_ (100 mL min^−1^) over Ni(10)–Ca(30)/Al_2_O_3_. As shown in Fig. 7, the concentration of uncaptured CO_2_ was approximately 350 ppm. The highest concentration of produced CH_4_ was 965 ppm; the concentrations of both the produced CO and desorbed CO_2_ were below 30 ppm. The conversion of captured CO_2_ and the selectivity for CH_4_ were 94% and 95%, respectively, indicating that Ni(10)–Ca(30)/Al_2_O_3_ favored the conversion of low concentrations of CO_2_. A long-term CCR operation experiment was also conducted using 300 mg of Ni(10)–Ca(30)/Al_2_O_3_ with flowing 1% CO_2_/10% O_2_/N_2_ for 24 h (Fig. 8). Initially, the produced CH_4_ concentration ranged from 3200 to 7800 ppm. After 24 h, the highest concentration of CH_4_ was 7400 ppm. The highest CO and uncaptured CO_2_ concentrations were below 150 and 250 ppm, respectively, for all the reaction times. The desorbed CO_2_ concentration ranged from 0 to 1500 ppm in the whole reaction process. Meanwhile, after reaction for 24 h, the CO_2_ conversion and CH_4_ yield were stable at 60% and 59%, respectively. These results indicate that the Ni(10)–Ca(30)/Al_2_O_3_ DFM not showed obvious deterioration in the CCR performance for at least 24 h, which exhibited a good durability. The effect of 20% water vapor on continuous CCR operation over Ni(10)–Ca(30)/Al_2_O_3_ was investigated to evaluate the potential for application to real natural gas power plant effluent (Fig. S4†). The uncaptured CO_2_ concentration was maintained between 1000 and 2000 ppm; however, the maximum concentration of formed CH_4_ decreased to 2700 ppm. Therefore, water removal from exhaust gas is necessary for real-world applications.

## Conclusion

Continuous CO_2_ capture and methanation reactions over Ni–Ca based DFMs were studied using double reactors in the presence of oxygen. The utilization of a double reactor system increased the amounts of CO_2_ captured and CH_4_ produced when compared to the performance of a single reactor system. A relatively high Ca loading (30 wt%) in the DFM was found to be the most effective for continuous CCR operation under isothermal conditions at 450 °C; thus, the Ni(10)–Ca(30)/Al_2_O_3_ sample displayed an excellent activity and good durability, maintaining its high CCR performance for at least 24 h. The XRD results revealed that a high Ca loading on Al_2_O_3_ induced the formation of Ca_12_Al_14_O_33_. Subsequent Ni loading resulted in the transformation of Ca_12_Al_14_O_33_ into amorphous structures, which was responsible for the favorable performance of Ni(10)–Ca(30)/Al_2_O_3,_ as indicated by TPD and TPSR measurements. The order of Ca and Ni introduction also affected the structure of the Ni–Ca DFM and its CCR performance, and better results were obtained when Ni was introduced last.

## Conflicts of interest

There are no conflicts to declare.

## Supplementary Material

RA-013-D2RA07554G-s001
