# Supplementary material for: Continuous CO2 capture and methanation over Ni–Ca/Al2O3 dual functional materials
Source: RSC Adv. 2023 Jan 12;13(4):2213–9. doi: 10.1039/d2ra07554g (PMC9835769; doi:10.1039/d2ra07554g)
Supplement: RA-013-D2RA07554G-s001 [file RA-013-D2RA07554G-s001.pdf]

Electronic Supplementary Material (ESI) for RSC Advances.
This journal is © The Royal Society of Chemistry 2022

Electronic supplementary information (ESI)

Continuous CO_2 capture and methanation over Ni-Ca/Al_2O_3 dual functional materials

Lingcong Li,[1] Ziyang Wu,[1] Shinta Miyazaki,[1] Takashi Toyao,[1] Zen Maeno,*[2] Ken-ichi Shimizu*[1]

[1] Institute for Catalysis, Hokkaido University, N-21, W-10, Sapporo 001-0021, Japan

[2] School of Advanced Engineering, Kogakuin University, 2665-1, Nakano-cho, Hachioji, 192-0015, Japan

*Corresponding authors

Zen Maeno, E-mail: zmaeno@cc.kogakuin.ac.jp

Ken-ichi Shimizu, E-mail: kshimizu@cat.hokudai.ac.jp

Figures

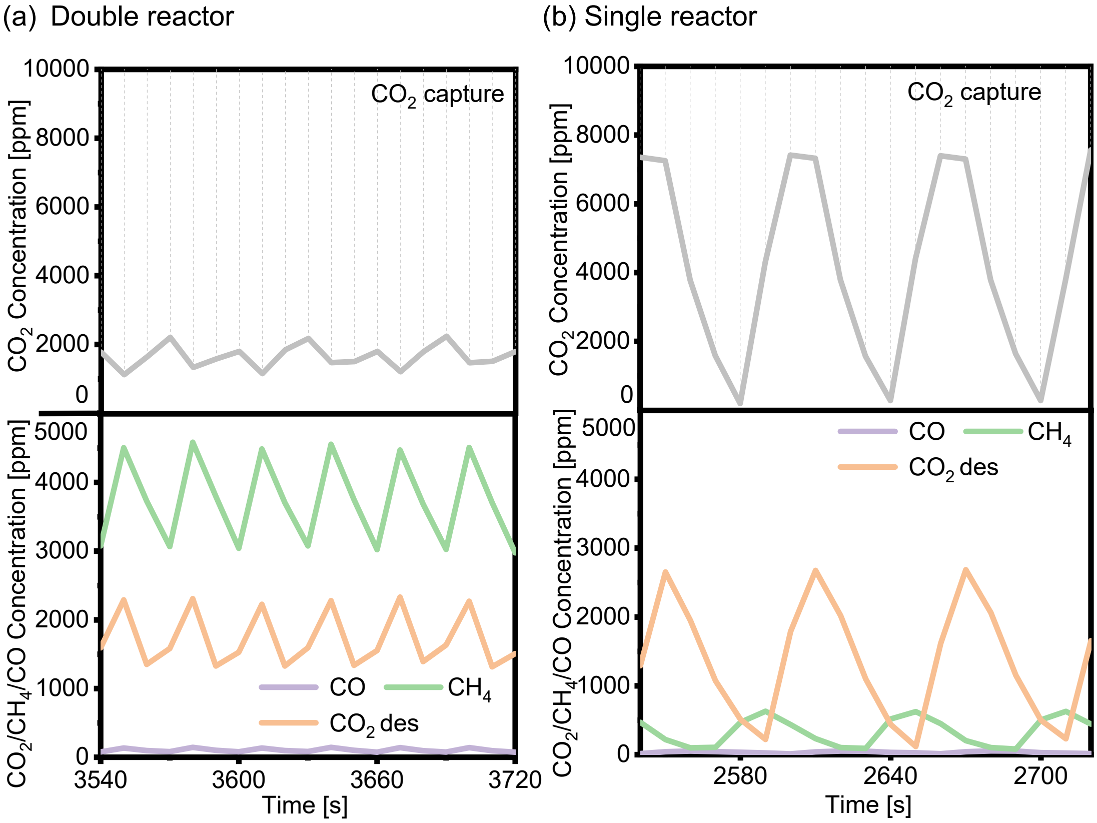

Figure S1. Effluent gas compositions for CCR over Ni(10)–Ca(30)/Al_2O_3 in (a) double reactor and (b) single reactor systems. Conditions: 100 mg of catalyst, 450 °C, 100 mL min^{-1} of 1% CO_2/10% O_2/N_2 for 30 s, switched to 100 mL min^{-1} of H_2 for 30 s.

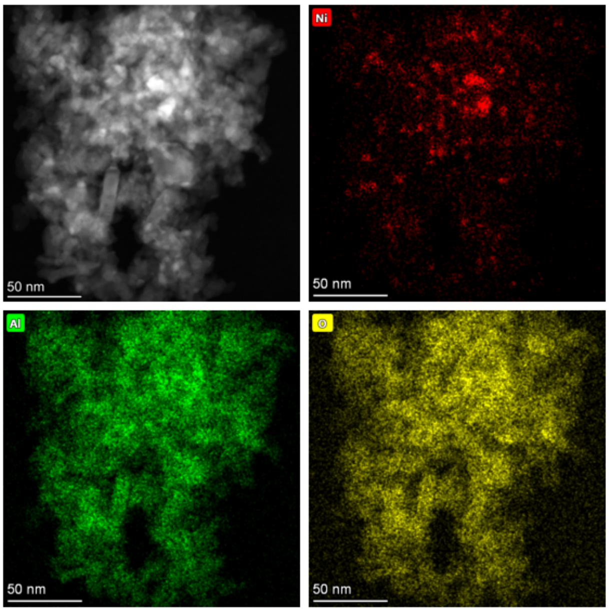

Figure S2. STEM images and EDS mapping of Ni(10)/Al_2O_3.

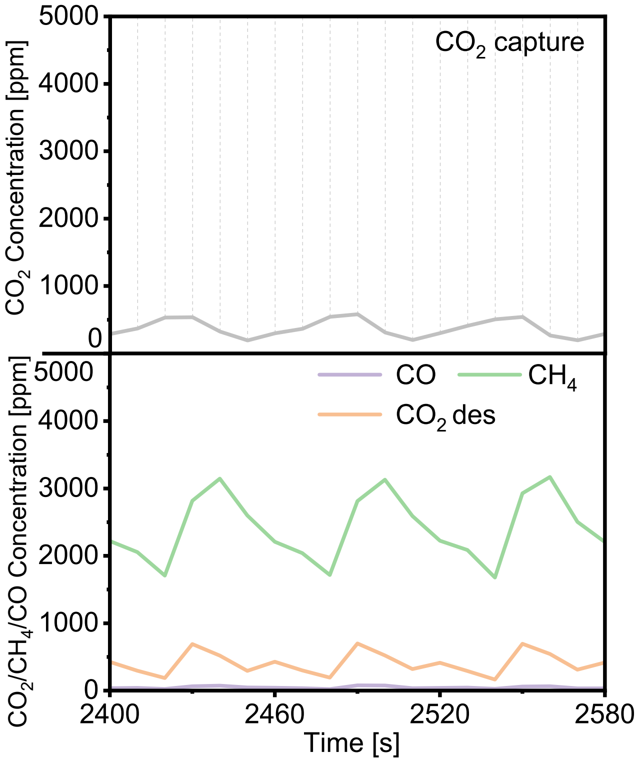

Figure S3 Effluent gas composition for continuous operated CCR over Ni(10)/$Ca_{12}Al_{14}O_{33}$. Conditions: 100 mg of catalyst, 450 °C, 100 mL min^{-1} of 0.5% CO_2/10% O_2/N_2 for 30 s, switched to 100 mL min^{-1} of H_2 for the other 30 s.

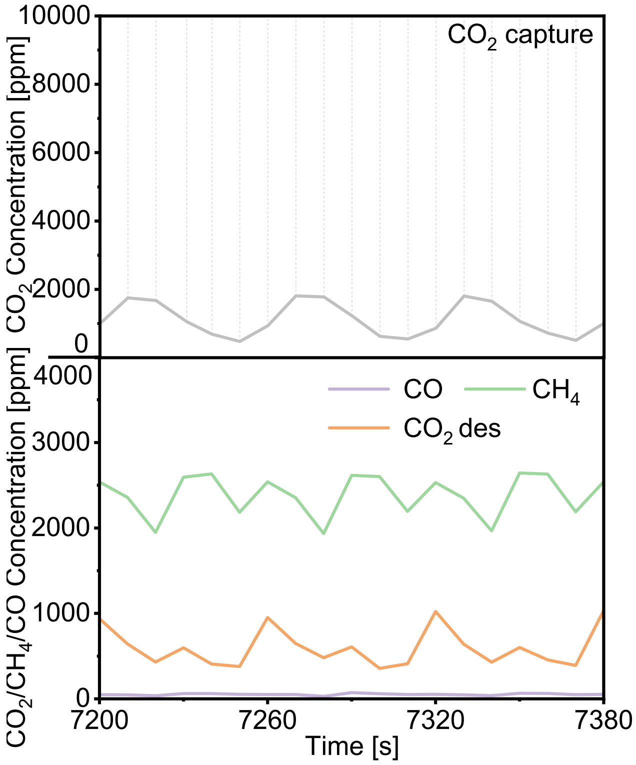

Figure S4 Continuous CCR operation with flowing steam over Ni(10)-Ca(30)/Al_2O_3. Conditions: 100 mg of catalyst for each reactor, 450 °C, 100 mL min^{-1} of 1% CO_2/10% O_2/N_2 with 20% water vapor for 30 s, switched to 100 mL min^{-1} of H_2 for the other 30 s.

Table S1. Comparison of CCR performance among the reported Ni-Ca based DFMs.

Ni-Ca DFM	Reaction Temperature [°C]	CO_2 capture [μmol g^{-1}]	CO_2 conversion (%)	CH_4 formation [μmol g^{-1}]	CH_4 selectivity (%)	Ref.
10% Ni-30% Ca/Al_2O_3	450	340	46	153	97	This work
1% Ni-CaO	550	9200	38	2000	58	Fuel **2021**, 286, 119308
10% Ni-CaO	550	8100	45	2500	69	Fuel **2021**, 286, 119308
10% Ni-15% Ca-Al_2O_3	450	73	82	58	97	*ACS Sustainable Chem. Eng.* **2021**, *9*, 3452–3463
5% Ni/15%Ca-Al_2O_3	520	----	----	223	----	*J. CO_2 Util.* **2019**, *34*, 576-587
10% Ni/15%Ca-Al_2O_3	520	----	----	225	----	*J. CO_2 Util.* **2019**, *34*, 576-587
15% Ni/20%Ca-Al_2O_3	450	----	66.6	----	63.2	*J. CO_2 Util.* **2019**, *31*, 143-151
